# An autophagy-related diagnostic biomarker for uterine fibroids: FOS

**DOI:** 10.3389/fmed.2023.1153537

**Published:** 2023-04-17

**Authors:** Lei Cai, Jie Li, Rui Long, Zhiqi Liao, Juejun Gong, Bowen Zheng, Hanwang Zhang

**Affiliations:** ^1^Reproductive Medicine Center, Tongji Hospital, Tongji Medical College, Huazhong University of Science and Technology, Wuhan, China; ^2^Department of Oncology, The Central Hospital of Wuhan, Tongji Medical College, Huazhong University of Science and Technology, Wuhan, China; ^3^Medical Record Department, Women and Children’s Hospital of Chongqing Medical University, Chongqing, China

**Keywords:** uterine fibroids, autophagy, FOS, bioinformatics analysis, biomarker

## Abstract

Uterine fibroids (UFs) are the most common benign gynecologic tumors in reproductive-aged women. The typical diagnostic strategies of UFs are transvaginal ultrasonography and pathological feature, while molecular biomarkers are considered conventional options in the assessment of the origin and development of UFs in recent years. Here, we extracted the differential expression genes (DEGs) and differential DNA methylation genes (DMGs) of UFs from the Gene Expression Omnibus (GEO) database, GSE64763, GSE120854, GSE45188, and GSE45187. 167 DEGs with aberrant DNA methylation were identified, and further Gene Ontology (GO) enrichment and Kyoto Encyclopedia of Genes and Genomes (KEGG) were performed by the relevant R package. We next discerned 2 hub genes (FOS, and TNFSF10) with autophagy involvement by overlapping 167 DEGs and 232 autophagic regulators from Human Autophagy Database. FOS was identified as the most crucial gene through the Protein–Protein Interactions (PPI) network with the correlation of the immune scores. Moreover, the down-regulated expression of FOS in UFs tissue at both mRNA and protein levels was validated by RT-qPCR and immunohistochemistry respectively. The area under the ROC curve (AUC) of FOS was 0.856, with a sensitivity of 86.2% and a specificity of 73.9%. Overall, we explored the possible biomarker of UFs undergoing DNA—methylated autophagy and provided clinicians with a comprehensive assessment of UFs.

## 1. Introduction

Uterine fibroids (UFs), also known as uterine leiomyoma, are the most common solid neoplasm in women with an estimated incidence of up to 70% (1). The established risk factors of UFs include increased age until menopause, premenopausal status, hypertension, obesity, or other chronic psychological stress, etc. (2–4). The symptomatic fibroids can manifest with prolonged or heavy menstrual bleeding and the sequelae of uterine enlargement, for instance, pelvic pressure, urinary frequency, and constipation, and it can be associated with infertility and other poor obstetrical outcomes (5). UFs caused the deterioration of the quality of life in women at reproductive age (6) and caused an extremely high economic burden on society (7, 8). Although transvaginal ultrasonography and pathological feature are the main diagnostic tools of UFs (9), molecular biomarkers are considered conventional strategies in the assessment of the origin and development of UFs in recent years (10). The highly prevalent condition of UFs restricted the biomarkers in a strict sensitivity and specificity to ensure their effectiveness. The efficacious biomarker should guarantee sensitivity >75% and specificity >99.6% (11). Thus, the accuracy biomarkers of UFs diagnosis still needed to be explored.

Autophagy is an evolutionarily conserved process that delivered a portion of the cytoplasm, such as ruptured lysosomes, intracellular microbes, and damaged mitochondria, into lysosomes for degradation via autophagosome formation (12). This process plays a crucial role in the pathogenesis of many diseases including uterine fibroids (13–15). The attenuation of autolysosomes in UFs tissue illustrated the defection of the fusion of the autophagosome with a lysosome in the late stages of autophagy (14). The primary uterine fibroids cells exhibited autophagic response after the stimulation with estradiol (E2) or ulipristal acetate, which is represented by required autophagy-related proteins (ATGs), MAP1LC3 (LC3), and P62, indicating that autophagy significantly involved in the pathophysiology of UFs (15–17). The regulation of autophagy is complex and dynamic, while epigenetics are considered to be the conspicuous machinery regulator of this process, particularly DNA methylation (18–20). DNA methylation is an important epigenetic mechanism of the transfer of a methyl (-CH3) group to the fifth carbon of a cytosine to form 5-methylcytosine (5mC) which induced the modification of gene expression (21). This process is generally presented as transcriptional silencing and occurs predominantly in cytosine guanine dinucleotide (CpG) dinucleotides (22). The genomic maps of DNA methylation, based on CpG site detection, provide information on regulatory regions of genes, those genes are functionally categorized in both ATGs and signal molecule genes that regulate autophagy (18).

The DNA methylation status of UFs is exhibited in the decreasing of DNA methyltransferases (DNMTs), subtypes DNMT3A (DNA methyltransferase 3 alpha) and DNMT3B (DNA methyltransferase 3 beta) (23). The genome-wide DNA methylation status in UFs tissue is distinguished from normal myometrium and the differential methylated genomic locus was also presented in UFs (24–27). The hypomethylated/hypermethylated genes are proven to participate in the proliferation, apoptosis, metabolism, and extracellular matrix formation of UFs (25). Nevertheless, whether the autophagic dysregulation in UFs is regulated by DNA methylation is still unknown.

In the present study, we extracted the hub genes in both differential expression and differential DNA methylation profiles in UFs from Gene Expression Omnibus (GEO) datasets. Identified the autophagic regulators from Human Autophagy Database throughout those hub genes. And the candidate was validated by further RT-qPCR, and immunohistochemistry. We aimed to explore the possible biomarker of UFs undergoing DNA-methylated autophagy, the diagnostic value was performed by the receiver operating characteristic (ROC) curve.

## 2. Materials and methods

### 2.1. Data collection

All datasets were downloaded from Gene Expression Omnibus (GEO) database^1^ with keywords: “uterine myoma,” “fibroid” or “leiomyoma,” and “DNA methylation.” The inclusion criteria included: (1) The organism was limited to UFs and normal myometrium. (2) All datasets were genome-wide gene expression profiles. (3) Case and control study. The exclusion criteria was another tissue. Four datasets (GSE64763, GSE120854, GSE45188, and GSE45187) were selected. Samples of UFs and normal myometrium were used for subsequent analysis. The gene expression profile and the genome-wide DNA methylation profile were extracted from GSE64763 and GSE120854 respectively as the discovery cohorts. And GSE45187 and GSE45188 were presented as the validation cohorts. The detailed information of all the datasets were summarized in Table 1.

**TABLE 1:** Gene Expression Omnibus (GEO) data sets.

Dataset	Organism	Platform	Data type	Sample type	Purpose
GSE64763	*Homo sapiens*	GPL571	Expression profiling by array	Uterine fibroid (*n* = 25)	Discovery cohort
				Normal myometrium (*n* = 29)	
GSE120854	*Homo sapiens*	GPL23976	Methylation profiling by array	Uterine fibroid (*n* = 24)	Discovery cohort
				Normal myometrium (*n* = 10)	
GSE45187	*Homo sapiens*	GPL13534	Methylation profiling by array	Uterine fibroid (*n* = 3)	Validation cohort
				Normal myometrium (*n* = 3)	
GSE45188	*Homo sapiens*	GPL6244	Expression profiling by array	Uterine fibroid (*n* = 3)	Validation cohort

### 2.2. Data processing

The “limma” package was used to analyze mRNA expression data and the “ChAMP” package was used to analyze DNA methylation data (28–30). All mRNA expression data were normalized by “normalizeBetweenArrays()” function. The DNA methylation expression data were normalized by “champ.norm()” function. The “pheatmap” package was used to cluster samples and discard outliers (Supplementary Figure 1). Outliers included GSM1579399 and GSM1579420 for mRNA, GSM3417163, GSM3417156, GSM3417160, GSM3417145, GSM417146, GSM417147, and GSM417148 for DNA methylation.

### 2.3. Identified the differentially expressed genes (DEGs)

The DEGs between the uterine fibroid and normal myometrium samples were identified using “limma” package (version 3.50.0) and the threshold for identifying DEGs was set to |log2fold change (log2FC)| > 1 and adjusted *P* value < 0.05 (30).

### 2.4. Identified differentially methylation genes (DMGs)

Identification of DMGs between uterine fibroid and normal myometrium was analyzed by “ChAMP” package (version 3.50.0) (29). The results of DMGs were filtered with |log2FC| > 0.1 and adjust *P* value < 0.05.

### 2.5. Gene Ontology (GO) and Kyoto Encyclopedia of Genes and Genomes (KEGG) enrichment

The GO and KEGG enrichment analysis of the DEGs were performed by the “clusterProfiler” (version 4.2.1) package (31). We filtered the results with a threshold set to *P* value < 0.05 and false discovery rate (FDR) < 0.05.

### 2.6. Protein–Protein Interaction network (PPI)

STRING^2^ is an online database for predicting interactions between proteins encoded by DEGs. We constructed the PPI network based on the STRING database and Cytoscape (version 3.8.2) software was used to visualize the results.

### 2.7. Estimation of stromal and immune scores

The scores of immune cells/stromal cells for the uterine fibroid and normal myometrium samples were calculated using the “ESTIMATE” package (version 1.0.13) based on the gene expression data extracted from GSE64763 dataset. Wilcoxon test was used to test the scoring results. The threshold was set to *P* < 0.05 as significant.

### 2.8. Relationship between key genes and immune status

The correlation coefficient between the key genes and the immune status for uterine fibroid and normal myometrium samples was calculated. Spearman correlation analysis was conducted after excluding the data from normal distribution. The statistical significance was set as *P* < 0.05.

### 2.9. Patients

This study included patients who were histologically diagnosed with uterine fibroids and underwent subsequent myomectomy or hysterectomy in Tongji Hospital from 2018 to 2022. The participants were excluded if they had been diagnosed with major medical problems, such as cardiovascular disease, diabetes, and autoimmune disease. The patients who were diagnosed with other gynecological diseases, such as adenomyosis, abnormal uterine bleeding, or cancers in the reproductive system were also excluded. They were also excluded if they were taking estrogen or progesterone before the surgery. Paired normal myometrium was biopsied at a distance of 2 cm from the fibroids. The basic information about the patients was obtained from the patient information management system of Tongji Hospital. The study was approved by the Ethics Committee of Tongji Medical College, Huazhong University of Science and Technology (2022S068).

### 2.10. RT-qPCR

Total RNA was isolated from UFs and normal myometrium tissue using RNA-easy Isolation Reagent (Vazyme, R701) according to the manufacturer’s instruction. Total RNA was converted to cDNA using PrimeScript™ RT Master Mix (Takara, RR036A). Then, real-time PCR analyses were carried out by using Taq Pro Universal SYBR qPCR Master Mix (Vazyme, Q712-02). The PCR primers were listed as follows: cFOS-F: GGGGCAAGGTGGAACAGTTAT, cFOS-R: CCGCTTGGAGTGTATCAGTCA, GAPDH-F: CTTG AATCGTTGTTGTTATG, GAPDH-R: ATGGTGGTATTTG TAGGC.

### 2.11. Immunohistochemistry

The 4μm thickness section of paraffin-embedded fibroids and myometrium tissue were deparaffinized and rehydrated using graded xylene and alcohol. The slides were boiled in Tris/EDTA buffer for the unmasking of the antigenic epitopes. Then the endogenous peroxidase activity was quenched by 10% H_2_O_2_. Goat serum was used to block for 30min, RT. The slides were then incubated with the primary antibody of cFOS (Abcam, ab222699, 1:400) overnight, then following by the HRP-labeled secondary antibody incubation the next morning after 3 times phosphate buffer saline with Tween 20 (PBST) washing. The 3,3-diaminobenzidine tetrahydrochloride (DAB) substrate-chromogen system was used to detect the peroxidase activity. The following calculation of all slides was derived from the previous report (32).

### 2.12. ROC curve analysis

The “pROC” package (version 1.18.0) was used for ROC curve analysis and the area under the curve (AUC) was used to estimate the diagnostic value of key genes. We verified the expression of key genes in samples at the DNA methylation level and mRNA expression level.

### 2.13. Statistical analysis

The statistical significance of differences between the two groups in Figures 5, 6 was analyzed using a *t*-test. *P* value less than 0.05 was considered significant. All analyses were conducted on R (version 4.1.2) and SPSS (version 24.0).

## 3. Results

### 3.1. Identification of DEGs and DMGs

**FIGURE 1:**
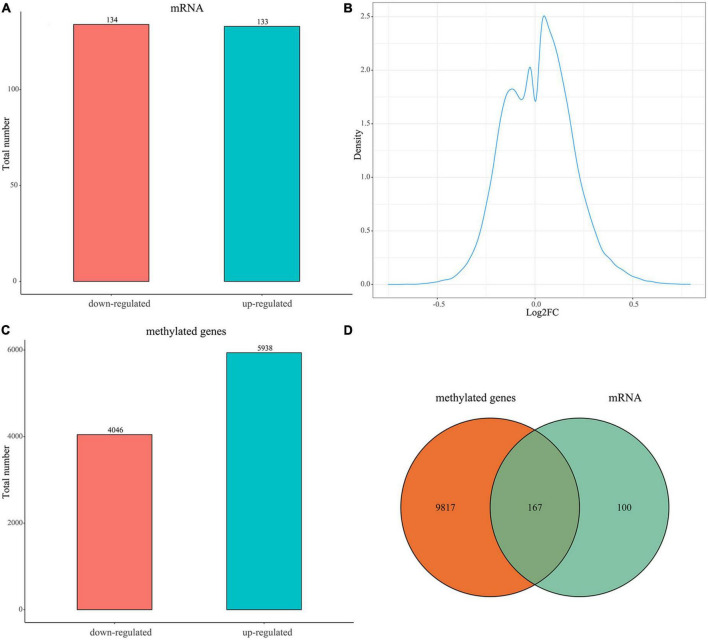
Expression of DEGs and DMGs between the uterine fibroid and normal myometrium tissue. **(A)** Bar plot for differentially expressed genes (DEGs) between the two groups. Red bar, down-regulated DEGs in UFs compared with NM; blue bar, up-regulated DEGs in UFs compared with NM. **(B)** Distribution characteristics of log2FC of differential methylation probes. The X-axis is the log2FC value of the differential methylation probe (UFs vs. NM). **(C)** Bar plot for differentially methylated genes (DMGs) between the two groups, Red bar, down-regulated DMGs in UFs; blue bar, up-regulated DMGs in UFs compared with NM. **(D)** Venn diagram of DMGs and DEGs, the overlapped part is used for further analysis. NM, normal myometrium, UFs, uterine fibroids.

A total of 267 DEGs between the uterine fibroid and normal myometrium tissue (133 up-regulated and 134 down-regulated) were identified in GSE64763 (Figure 1A and Supplementary Table 1). Differentially methylation probes were identified under the threshold of |log2FC| > 0.1 (Figure 1B). Among them, 4,046 hypomethylation genes and 5938 hypermethylation genes were extracted (Figure 1C). There were 167 genes overlapped in DEGs and DMGs (Figure 1D and Supplementary Table 1). We mainly carried out the follow-up analysis on these 167 genes.

### 3.2. Enrichment of methylation related genes (MRGs)

**FIGURE 2:**
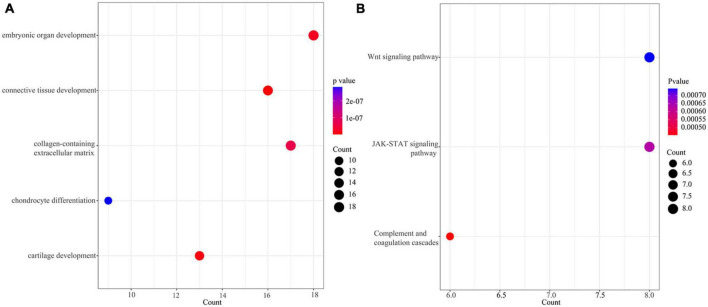
Enrichment of methylation related genes (MRGs). **(A)** The top 5 terms (ordered from small to large by FDR) of GO enrichment analysis. **(B)** KEGG of MRGs.

The extracted 167 DEGs with distinct methylation levels were defined as methylation related genes (MRGs). We performed functional enrichment analysis on 167 MRGs. Figure 2A showed the top five terms (ordered by FDR) of GO enrichment analysis (Supplementary Table 2). A total of three pathways were enriched under the KEGG analysis, including the “Wnt signaling pathway,” “JAK-STAT signaling pathway,” and “Complement and coagulation cascades” (Figure 2B and Supplementary Table 2).

### 3.3. Autophagy and PPI

**FIGURE 3:**
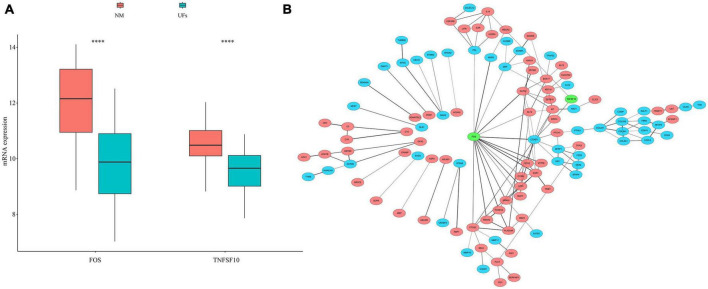
Autophagy genes in methylation related genes (MRGs). **(A)** The expression differences of autophagy gene in MRGs. **(B)** PPI network of MRGs and the position of autophagy gene in the network. Red marks down-regulated MRGs, blue marks up-regulated MRGs, and green marks the location of autophagy genes. NM, normal myometrium, UFs, uterine fibroids. ^****^P < 0.0001.

We extracted 232 autophagy related genes from the autophagy website^3^ (Supplementary Table 3), then overlapped 167 MRGs with those 232 autophagy related genes, FOS and TNFSF10 were identified. The mRNA expression of FOS and TNFSF10 in the UFs and myometrium was verified based on the normalized datasets. As shown in Figure 3A, both the FOS and TNFSF10 expression was down-regulated in the UFs group compared with the normal myometrium. The PPI network was visualized based on 167 MRGs with the combined scores of every node restricted over 0.5 (Supplementary Table 4). As shown in Figure 3B, the green-marked FOS and TNFSF10 were illustrated, and FOS connected with more complex interaction network than TNFSF10. Therefore, further analysis was presented with FOS priority.

### 3.4. Estimation of stromal and immune scores

**FIGURE 4:**
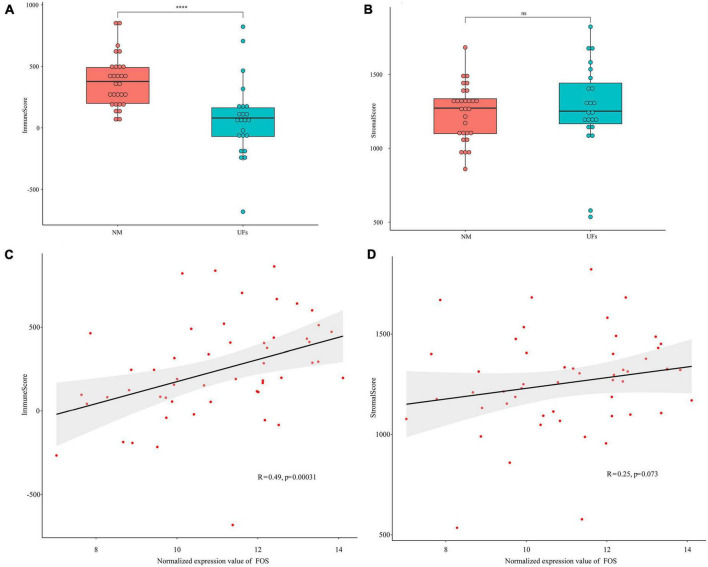
Estimation of stromal and immune scores between the fibroid and normal myometrium tissue. **(A)** Immune scores in the two groups. **(B)** Stromal scores in the two groups. **(C,D)** Correlation between FOS and stromal scores as well as immune scores. ^****^P < 0.0001.

The stromal and immune scores were further estimated based on the extracted dataset. The immune scores of uterine fibroid samples were significantly lower than that of normal myometrium (Figure 4A), while the stromal scores showed no significant difference between UFs and myometrium (Figure 4B). FOS presented a correlation with immune scores and the immune scores were raising up along with the increase in FOS expression level (Figure 4C). The stromal scores presented no correlation with the FOS expression according to the spearman analysis (Figure 4D).

**FIGURE 5:**
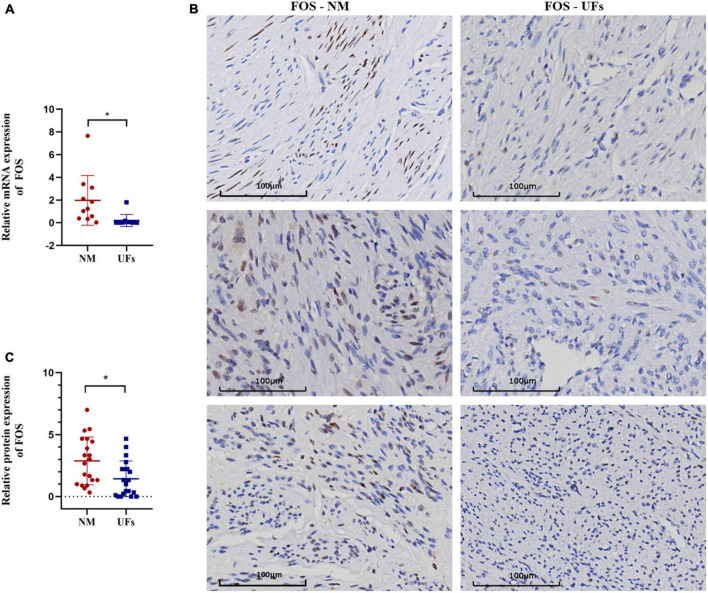
The expression of FOC in fibroid and normal myometrium tissue. **(A)** The relative expression of FOS in the mRNA level. **(B)** Immunohistochemical results of typical samples from two groups, all images were presented at 20-X. **(C)** IHC score. NM, normal myometrium, UFs, uterine fibroids. *P < 0.05.

**FIGURE 6:**
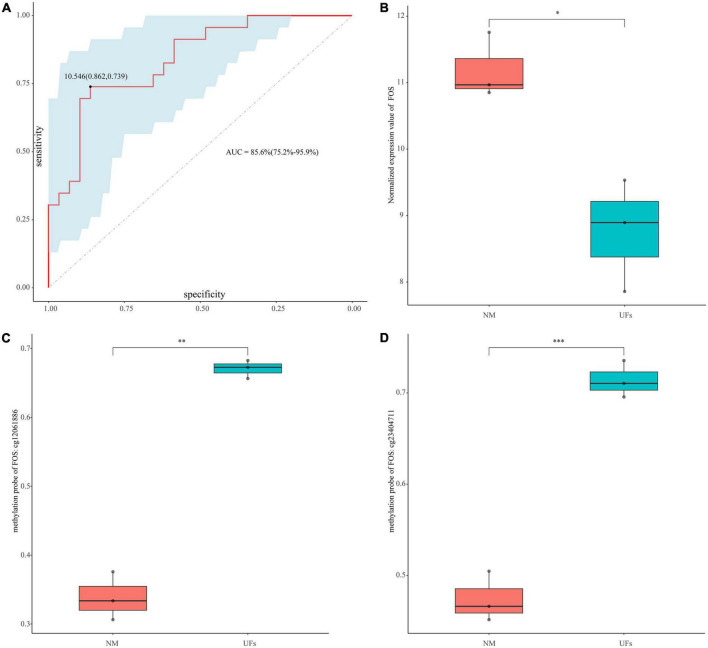
Diagnostic value of FOS. **(A)** ROC curve analysis and area under curve (AUC) of FOS. **(B)** GSE45188 supported the low expression of FOS in the fibroid samples. **(C,D)** The methylation level of the two differential methylation probes of FOS in GSE45188. *P < 0.05, ^**^P < 0.01, and ^***^P < 0.001.

### 3.5. Baseline characteristics of the patients

The characteristics of the total of 20 recruited patients were presented in Table 2. The mean age of the patients (±standard deviation) was 44.2 ± 5.75 years, ranging from 31 to 56. The 7(35%) of fibroids were located in the anterior of the uterine in this study. The maximum diameter of fibroids was less than 8 cm in most of the patients (18/20). Most of the patients (18/20) had no history of myomectomy before.

**TABLE 2:** Baseline characteristics of the study patients (*n* = 20).

Parameters		No. cases (%)
Age	<40	3 (15%)
	40–45	9 (45%)
	>45	7 (35%)
Location	Anterior	7 (35%)
	Posterior	6 (30%)
	Lateral	2 (10%)
	Fundal	2 (10%)
	others	3 (15%)
Maximum diameter	<5	8 (40%)
	5–8	9 (45%)
	>8	2 (10%)
Previous pregnancies	0	3 (15%)
	1–2	9 (45%)
	>2	7 (35%)
Previous myomectomy	Yes	2 (10%)
	No	18 (90%)

### 3.6. The expression of FOS in fibroids and normal myometrium from UFs patients

The expression of FOS was investigated using real-time quantitative PCR and IHC in the fibroid and paired myometrium from 20 UFs patients. As shown in Figure 5, FOS was downregulated in the fibroid tissue compared with the normal in both mRNA (Figure 5A) and protein levels (Figure 5B, C). Compared with the partial positive of FOS in the myometrium, fibroid tissue was nearly negative in FOS, only a minority week-stained cell could be captured in the IHC slice, and the IHC score of all samples was shown in Figure 5C.

### 3.7. Diagnostic value

The diagnosis model of FOS was built based on GSE64763. AUC was 0.856 (95% confidence interval: 75.2–95.9%), and the sensitivity and specificity were 0.862 and 0.739, respectively (Figure 6A). GSE45188 was used as a validation cohort to support the low expression of FOS in the fibroid samples (Figure 6B and Supplementary Table 5). Figures 6C, D showed the differential methylation probes of FOS, indicating that FOS was in the hypermethylation state in the fibroid samples (Supplementary Table 5).

## 4. Discussion

Uterine fibroids (UFs) are regarded as the most common pelvic tumors in women of childbearing age and usually cause heavy menstrual bleeding, pain, and infertility. Although previous studies have demonstrated the potential biomarkers for the origin and development of UFs, the efficacies were still unclear. In this study, FOS was identified as a potential biomarker as well as a possible molecular mechanism underlying the development of UFs by comprehensively analyzing multiple databases and validating the down-regulated expression of FOS in UFs tissue by IHC and RT-PCR.

It has been widely recognized that aberrant DNA methylation is significantly associated with UFs. Several studies demonstrated that the aberrant DNA methylation of the key tumor suppressor and developmental genes may partly involve in the pathogenesis of UFs via genome-wide DNA methylation assays and *in vitro* experiments (24). Therefore, in the current study, we analyzed the overlapped DEGs and DMGs of 4 datasets, including GSE64763, GSE120854, GSE45188, and GSE45187. A total of 167 DEGs with aberrant DNA methylation were identified between UFs and normal myometrium tissue samples. According to further GO and KEGG analysis, the DEGs were mainly enriched in connective tissue development and collagen-containing extracellular matrix, as well as the Wnt signaling pathway and JAK-STAT signaling pathway. The GO enrichment results are in line with our common experiments since UFs are composed of smooth muscle cells and varying amounts of fibrous connective tissue (33). The wingless-type (Wnt) signaling is considered a growth and development-related factor of the UFs, the elevated expression of WNT11, WNT16, and WNT5b, etc., were widely reported (34, 35). Canonical Wnt signaling pathway inhibitors reduce the proliferation of the primary human UFs cells and especially in the MED12 mutations type UFs which could be found in 70% of the UFs (36–38). Dai and his colleagues found that the promotion of uterine fibroids cell proliferation was accompanied by an increase in STAT-3 protein expression (39). Those studies supported our analyzed results that Wnt and JAK-STAT signaling pathways were involved in the development of UFs.

Aautophagy is a key contributor to the pathogenesis of UFs. In the Andaloussi AE et al. study, dysregulated autophagy has been shown to promote the growth of UFs in humans (14). Potential biomarkers of UFs collaborative diagnosis may be explored from the aspect of DNA methylation and autophagy. Therefore, 2 hub genes (FOS, and TNFSF10) with autophagy involvement were discerned from the overlapping of 167 DEGs and the aberrantly autophagic genes extracted from the Human Autophagy Database. According to the PPI network with the more complicated interaction networks, FOS was speculated as a crucial gene in the molecular mechanism underlying the development of UFs.

The FOS gene encodes for a protein that contains a leucine zipper and dimerizes the activator protein 1 (AP1) complex which works as a transcription factor with the JUN family (40). The FOS protein has been widely reported in several cancers and inflammatory diseases as a regulator of cell proliferation, differentiation, and transformation (41). However, the relevant studies on the aspect of UFs were limited. The reduction of FOS in mRNA transcripts has been reported by Mark Payson et al. by RT-PCR in UFs compared with myometrium (42), and the decreasing of FOS has been reported to be impervious to the different menstrual cycle phases or GnRHa treatment (43). The reduction protein level of FOS was reported by Lessl M et al. which consists of our results (44). In the current study, we first extracted FOS as a potential biomarker of UFs by comprehensive analysis of autophagy and DNA methylation related genes, which inspired us to that the origin of UFs may consist of both impaired autophagy and DNA methylation with the down-regulation of FOS. We further validated the decreased expression of FOS in UFs tissue at both mRNA and protein levels by the tissue samples from Asian females.

Immune and inflammation play important roles in the pathophysiology of the UFs. The peripheral immune cell presented diverse conditions in the UFs patients, for instance, circulating CD4/CD8 T cells were increased while NK cells were decreased (45). Several studies highlighted the involvement and importance of the macrophages in the inflammation and consequent fibrosis which are typical features of UFs tissue (46). The study of indicated a higher level of macrophage infiltration in the myoma nodules and the autologous endometrium of the submucosal myomas (SMM) and intramural myomas (IMM) compared with women without UFs (47). In the present study, we estimated the immune scores of FOS in UFs patients, the positive correlation of the immune scores and the FOS expression indicated that autophagic-related mechanism was not the unique pathophysiologic prospect of the UFs, the FOS-related immune disorder may also involve in this process.

FOS is considered one of the diagnostic biomarkers of UFs which presented with decreased expression in UFs tissue. The diagnostic value of FOS was verified via AUC with a sensitivity of 86.2% and a specificity of 73.9%. However, the limitation is that the diagnostic value of FOS in UFs still based on invasive hysterectomy or myomectomy. The present study proposed the hypothesis of the FOS involved mechanisms of UFs development which is anomalous DNA methylation and autophagy condition, even the concomitant immune disorder.

## 5. Conclusion

In conclusion, we identified FOS as an autophagy-related biomarker for UFs by the comprehensive analysis of differential expression genes with aberrant DNA methylation and autophagy-related genes. And we validated the down-regulation of FOS in UFs tissue. These findings may reveal a potential diagnostic biomarker of uterine fibroids.

## Data availability statement

The datasets presented in this study can be found in online repositories. The names of the repository/repositories and accession number(s) can be found in the article/Supplementary material.

## Ethics statement

The studies involving human participants were reviewed and approved by the Ethics Committee of Tongji Medical College, Huazhong University of Science and Technology. The patients/participants provided their written informed consent to participate in this study.

## Author contributions

HZ: study conception. LC: research design, UFs, and normal myometrium sample collection, manuscript preparation, conduction of the RT-PCR, and IHC experiments. JL: research design, data acquisition, and manuscript preparation. RL and JG: research design and manuscript preparation. ZL and BZ: check manuscript. All authors approved the final version to be published.

## Footnotes

1
https://www.ncbi.nlm.nih.gov/geo/

2
https://cn.string-db.org/

3
http://www.autophagy.lu/index.html
