# Supplementary figures and images for: An autophagy-related diagnostic biomarker for uterine fibroids: FOS
Source: Front Med (Lausanne). 2023 Apr 17;10:1153537. doi: 10.3389/fmed.2023.1153537 (PMC10150886; doi:10.3389/fmed.2023.1153537)

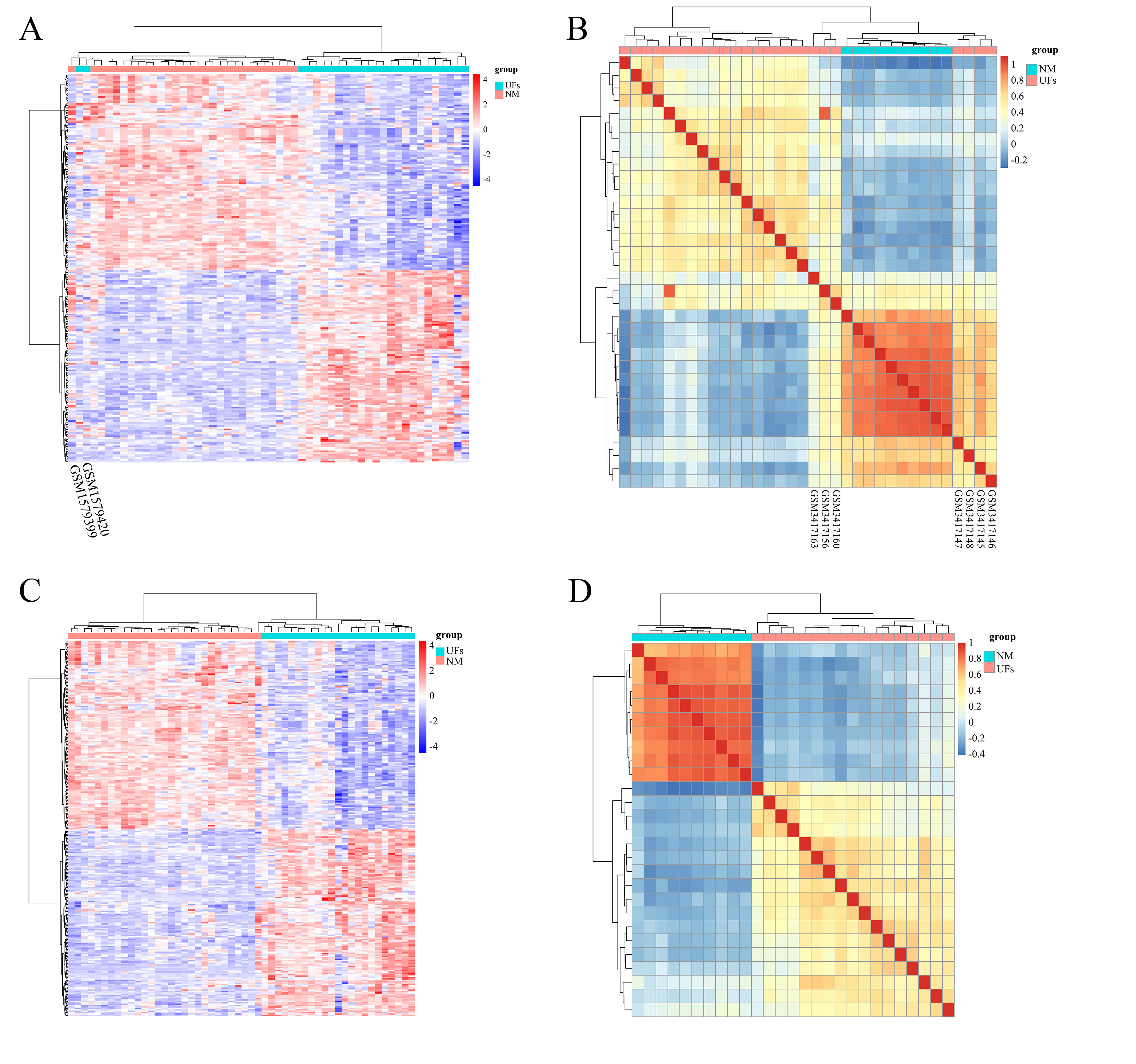

Supplement: Supplementary file 1 [file Image_1.JPEG]
